# Repurposing Modular Polyketide Synthases and Non-ribosomal Peptide Synthetases for Novel Chemical Biosynthesis

**DOI:** 10.3389/fmolb.2020.00087

**Published:** 2020-05-15

**Authors:** Soonkyu Hwang, Namil Lee, Suhyung Cho, Bernhard Palsson, Byung-Kwan Cho

**Affiliations:** ^1^Systems and Synthetic Biology Laboratory, Department of Biological Sciences and KI for the BioCentury, Korea Advanced Institute of Science and Technology, Daejeon, South Korea; ^2^Department of Bioengineering, University of California, San Diego, La Jolla, CA, United States; ^3^Department of Pediatrics, University of California, San Diego, La Jolla, CA, United States; ^4^The Novo Nordisk Foundation Center for Biosustainability, Technical University of Denmark, Lyngby, Denmark; ^5^Intelligent Synthetic Biology Center, Daejeon, South Korea

**Keywords:** polyketide synthase, non-ribosomal peptide synthetase, domain, module, repurposing

## Abstract

In nature, various enzymes govern diverse biochemical reactions through their specific three-dimensional structures, which have been harnessed to produce many useful bioactive compounds including clinical agents and commodity chemicals. Polyketide synthases (PKSs) and non-ribosomal peptide synthetases (NRPSs) are particularly unique multifunctional enzymes that display modular organization. Individual modules incorporate their own specific substrates and collaborate to assemble complex polyketides or non-ribosomal polypeptides in a linear fashion. Due to the modular properties of PKSs and NRPSs, they have been attractive rational engineering targets for novel chemical production through the predictable modification of each moiety of the complex chemical through engineering of the cognate module. Thus, individual reactions of each module could be separated as a retro-biosynthetic biopart and repurposed to new biosynthetic pathways for the production of biofuels or commodity chemicals. Despite these potentials, repurposing attempts have often failed owing to impaired catalytic activity or the production of unintended products due to incompatible protein–protein interactions between the modules and structural perturbation of the enzyme. Recent advances in the structural, computational, and synthetic tools provide more opportunities for successful repurposing. In this review, we focused on the representative strategies and examples for the repurposing of modular PKSs and NRPSs, along with their advantages and current limitations. Thereafter, synthetic biology tools and perspectives were suggested for potential further advancement, including the rational and large-scale high-throughput approaches. Ultimately, the potential diverse reactions from modular PKSs and NRPSs would be leveraged to expand the reservoir of useful chemicals.

## Introduction

Enzymes are biosynthetic protein machineries that recognize specific substrates through unique three-dimensional structures, and catalyze the conversion of these substrates into new biomolecules (Agarwal, 2006). Harnessing their diverse biochemical reactions has led to the production of many bioactive-compounds as clinical agents and commodity chemicals (Tibrewal and Tang, 2014). In addition, engineering such enzymes and repurposing their reactions into new pathways enhances the biocatalytic properties and the diversity of the natural products, respectively (Tibrewal and Tang, 2014). Biosynthesis of organic compounds has several advantages compared to classical chemical synthesis methods (Wenda et al., 2011). First, enzymes are not environmentally harmful, they act as non-toxic catalysts. The reaction conditions for the production of diverse chemicals are generally moderate in terms of temperature, pressure, and pH, while the classical chemical synthesis often requires extreme conditions. High selectivity of enzymes yields high purities with specific stereochemistry of the product and reduce undesired by-products and toxic intermediates. In nature, interestingly, the mechanisms underlying a large number of enzyme reactions have not been discovered yet. For example, the recent genome mining efforts on bacteria, fungi, and plants have revealed the richness of secondary metabolite biosynthetic gene clusters (smBGCs) including many unidentified smBGCs (Rutledge and Challis, 2015). Thus, novel non-natural chemicals and pathways have been constructed by the reprogramming of the multi-enzyme complex encoded by smBGCs (Bernhardt and O’Connor, 2009; Chevrette et al., 2019).

Type I modular polyketide synthases (PKSs) and non-ribosomal peptide synthetases (NRPSs) are prominent engineering targets due to their modular properties of enzyme assembly (Ladner and Williams, 2016). Type I modular PKS comprises several modules, each responsible for the incorporation and modification of one acyl-CoA substrate to synthesize the polyketide product, such as erythromycin (antibiotic), rapamycin (immunosuppressant), amphotericin B (antifungal), and other potential clinical agents (Staunton and Weissman, 2001). Likewise, NRPS comprises numerous modules, with each of them responsible for the incorporation and modification of one amino acid substrate to extend the polypeptide product, such as daptomycin (antibiotic), actinomycin D (antitumor), cyclosporine A (immunosuppressant), and other potential clinical agents (Walsh, 2016; Sussmuth and Mainz, 2017). As the organization and the order of these modules are co-linearly correlated with each unit of the final polyketide and polypeptide products, the target modules of rational engineering could be predicted for the production of novel derivatives having the modified unit at a specific position (Alanjary et al., 2019). Using this modular biosynthetic logic of type I modular PKS and NRPS, each reaction of a module was capable of being separated from the original assembly reactions and repurposed to construct *de novo* biosynthetic pathway for other chemicals (Pang et al., 2019). This approach is favorable in terms of (i) the potential diversity of available synthetic parts governing unique chemical reactions, (ii) enabling retro-biosynthesis by combinatorial assembly of domains and modules, and (iii) the relative ease of engineering, owing to avoidance of the structural perturbation compared to the engineering within the multi-enzyme complex.

In this review, we briefly introduced the structure and mechanism of the modular PKS and NRPS, and thereafter focused on their repurposing examples, along with their advantages and limitations. Finally, tools in the design-build-test-learn cycle of synthetic biology and the future perspectives of the repurposing strategies were discussed.

## Modular PKS and NRPS Architecture and Mechanism

**FIGURE 1:**
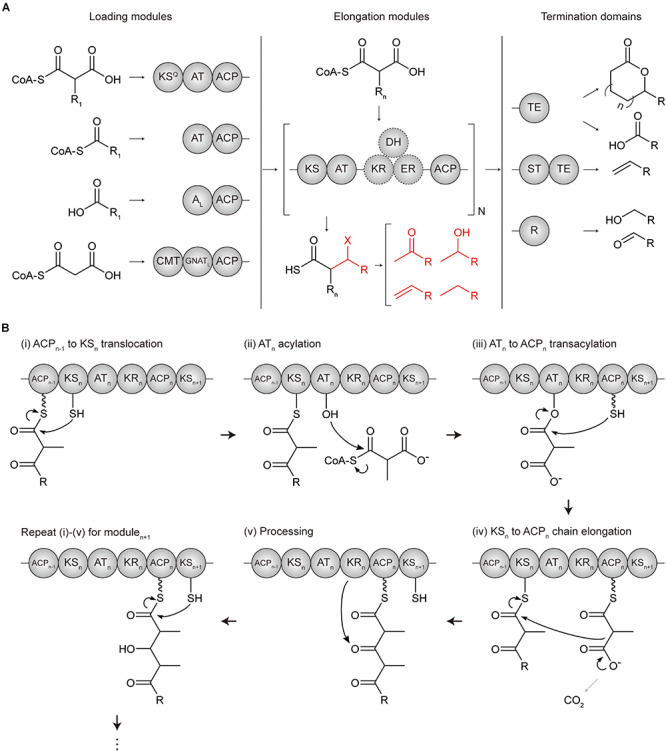
Domain architectures and mechanisms of polyketide chain extension in modular PKS. **(A)** Overall flow scheme of polyketide biosynthesis with different domain architectures of modules. Four types of loading modules load the different substrates according to involved domains (chemical examples were indicated). Next, the extender unit is selected and condensed to the growing chain one by one per elongation module for N cycles. Optional reductive domains (dashed circles) reduce the β-carbon group resulting in different X groups (indicated in red). Finally, the growing polyketide chain is cleaved by three different types of offloading domains in termination modules producing different products, including linear carboxylic acids, macrocyclic acids, olefins, aldehydes, and primary alcohols. **(B)** Mechanism of polyketide chain extension for the elongation module_*n*_. (i) ACP_*n*__–__1_ to KS_*n*_ translocation; the active site cysteine moiety of KS_*n*_ receives the growing polyketide chain of ACP_*n*__–__1_. (ii) AT_*n*_ acylation; the cognate acyl unit is incorporated into the active site serine moiety of ATn to form the acyl-*O*-AT intermediate. (iii) AT_*n*_ to ACP_*n*_ transacylation; the acyl group of AT_*n*_ is transacylated to the ACP_*n*_. (iv) KS_*n*_ to ACP_*n*_ chain elongation; KS_*n*_ catalyzes a decarboxylative Claisen condensation between the growing polyketide chain and the acyl extender unit of ACP_*n*_ for the chain extension. (v) Processing; the extender units of ACP_*n*_ are modified by a reductive loop or other additional domains. ACP, acyl carrier protein; A_L_, CoA ligase-type domain; AT, acyltransferase; CMT, *C*-methyltransferase; GNAT_*L*_, GCN5 *N*-acetyltransferase-like domain; DH, dehydratase; ER, enoylreductase; KR, ketoreductase; KS, ketosynthase; KS^*Q*^, condensation-incompetent ketosynthase; R, reductive domain; ST, sulfotransferase; TE, thioesterase.

Polyketide synthases are categorized into three types, namely, types I, II, and III, according to their organization and catalytic mechanisms (Yu et al., 2012). Among them, type I modular PKS has a hierarchical organization in which, the entire enzyme complex is composed of several subunits, each subunit is composed of several modules, and a module is composed of several domains (Bayly and Yadav, 2017) (Figure 1A). A minimal elongation module includes three domains; (i) an acyltransferase (AT) domain for loading the chain extender unit (typically malonyl- or methylmalonyl-CoA), (ii) an acyl carrier protein (ACP) for tethering and shuttling the extender unit or the polyketide intermediate, and (iii) a ketosynthase (KS) domain for catalyzing the condensation reaction between the extender unit of the downstream ACP domain and the polyketide intermediate attached at the KS active site which is translocated from the ACP domain of upstream module. Addition of other domains to this minimal elongation module modifies a polyketide backbone.

In the N- to C-terminus of a whole enzyme complex, loading, elongation, and termination modules are localized to catalyze the serial polyketide production (Barajas et al., 2017). Loading module (LM) initiates the chain formation from a broad range of priming starter units by acylation to the AT domain and transacylation to the ACP domain. According to the configuration of the domains, LMs are divided into subtype A, B, C, and D (Kornfuehrer and Eustáquio, 2019). In addition to AT-ACP didomain, type A LMs involve the condensation-incompetent KS domain that decarboxylates malonyl- or methylmalonyl-CoA to yield acetyl- or propionyl starter units, respectively. Type B LM consist of only the AT-ACP didomain and has a much broader range of substrates. Type C LM has a CoA-ligase domain rather than AT domain to incorporate carboxylic acid substrate in an ATP-dependent manner. Lastly, type D LM has a GCN5 *N*-acetyltransferase-like domain rather than AT domain that recently repurposed to catalyze decarboxylation (Skiba et al., 2020). A common characteristic of LMs is the absence of the condensation domain, which results a more flexible substrate specificity of the AT domain than those of downstream modules. Elongation modules consist of KS, AT, ACP, and other additional domains to serially catalyze the growth of the polyketide chain (Barajas et al., 2017; Zargar et al., 2018; Kornfuehrer and Eustáquio, 2019) (Figure 1B). Termination module contains a thioesterase (TE) domain or reductive (R) domain as an offloading domain following the ACP domain to release the ACP-bound polyketide intermediate and complete polyketide biosynthesis. TE domain performs; (i) hydrolysis to yield the linear polyketide, or (ii) cyclization to yield the macrocyclic polyketide, or (iii) sulfonate transfer and decarboxylation with sulfotransferase (ST) domain to yield the terminal olefin. Alternatively, R domain catalyzes NADPH dependent two-electron reduction, yielding the aldehyde product.

**FIGURE 2:**
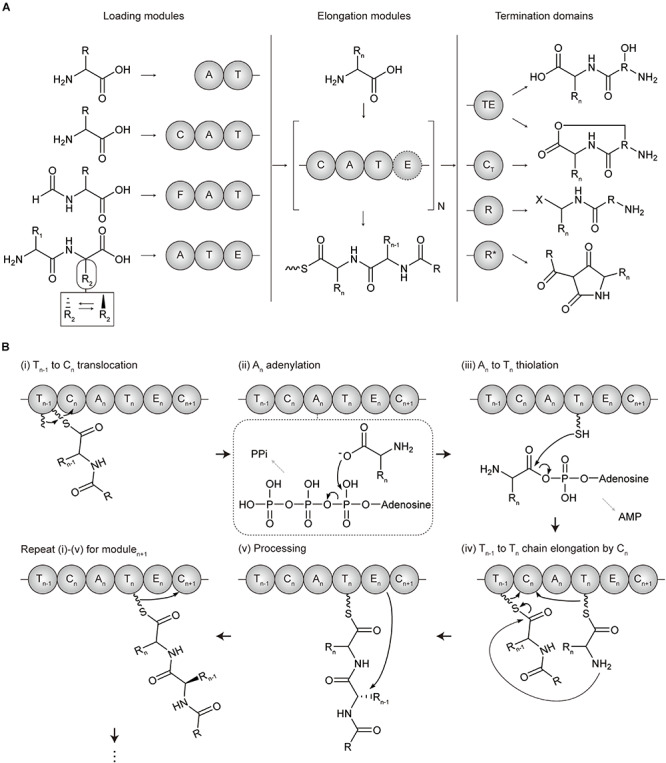
Domain architectures and mechanisms of non-ribosomal peptide chain extension in modular NRPS. **(A)** Overall flow scheme of non-ribosomal peptide biosynthesis with different domain architectures of modules. Four representative types of loading modules load the different substrates according to the involved domains (chemical examples were indicated). Next, the extender unit is selected and condensed to the growing chain one by one per elongation module for N cycles. An example of optional processing domain is indicated by the dashed circles. Finally, the growing non-ribosomal peptide chain is cleaved by four representative types of offloading domains in termination modules, producing different products including linear peptides, macrocyclic peptides, aldehydes, and tetramate moieties. The terminal X group of the product from terminal R domain includes hydroxyl group (-OH), aldehyde group (-CHO), and other aldehyde derivatives (Barajas et al., 2015; Dan et al., 2019). **(B)** Mechanism of polyketide chain extension for the elongation module_*n*_. (i) *T*_*n*__–__1_ to *C*_*n*_ translocation; the growing non-ribosomal peptide chain linked to Ppant arm of *T*_*n*__–__1_ domain translocates to the solvent channel of *C*_*n*_ domain donor site. (ii) *A*_*n*_ adenylation; the extender amino acid unit is activated by ATP to form aminoacyl-AMP in *A*_*n*_ domain. (iii) *A*_*n*_ to *T*_*n*_ thiolation; the aminoacyl-AMP intermediate of *A*_*n*_ is transferred to the Ppant arm of *T*_*n*_ domain to form aminoacyl thioester intermediate. (iv) *T*_*n*__–__1_ to *T*_*n*_ condensation at *C*_*n*_; the aminoacyl thioester intermediate of *T*_*n*_ domain is translocated to the solvent channel of *C*_*n*_ domain acceptor site, and the peptide bond formation between the growing peptide of *T*_*n*__–__1_ domain and the amino acid extender unit of *T*_*n*_ domain elongates by adding one amino acid to the growing peptide. (v) Processing; the extender units of ACP_*n*_ are modified by an epimerase (E) domain or other additional domains. A, adenylation domain; C, condensation domain; C_*T*_, terminal condensation domain; F, formylating domain; R, reductive domain; R*, R-like domain; T, thiolation domain; TE, thioesterase.

Non-ribosomal peptide synthetases are categorized into two types according to their organizations and catalytic mechanisms, which are type I modular NRPS and type II standalone NRPS (Jaremko et al., 2019). Type I modular NRPS a resemblance to type I modular PKS, with a hierarchical organization of the enzyme complex-subunit-module-domain (Winn et al., 2016) (Figure 2A). A minimal elongation module includes three domains which are, an adenylation (A) domain for loading the amino acid extender unit (both proteinogenic and non-proteinogenic), an thiolation (T or PCP) domain for tethering and shuttling the extender unit transferred from the upstream A domain or the growing polypeptide transferred from T domain of the upstream module, and a condensation (C) domain for catalyzing the peptide bond formation between the amino acid extender unit of the downstream T domain and the growing polypeptide of the T domain of the upstream module. Other *cis*-acting and *trans*-acting domains to this minimal elongation module expand the extender amino acid unit.

Similar to PKS, loading, elongation, and termination module are localized to catalyze serial polypeptide production (Sussmuth and Mainz, 2017). NRPS LMs usually lack a C domain and are more variable than PKS. Many NRPS LMs carry the N-terminal modifications, which function as protection against degradation, modulating polarity, and providing specific properties such as membrane insertion. Several additional domains including formylating domain, CoA ligase domain, and other tailoring domains could be involved in LMs for acylation and formylation, among others. NRPS elongation module basically consists of three core domains C, A, and T corresponding to KS, AT, and ACP of PKS which play a role in chain elongation, substrate incorporation, and chain carrier function, respectively (Hur et al., 2012; Winn et al., 2016; Bloudoff and Schmeing, 2017; Sussmuth and Mainz, 2017) (Figure 2B). However, their overall structures and mechanisms are quite different, and there are also more various and distinct processing domains for NRPS. Examples include; epimerase (E) domain catalyzing the absolute configuration at the C_α_ atom, methyltransferse (MT) domain modifying the degree of C- or N-methylation, formylation (F) domain, cyclization (Cy), redox-active domain (Ox and Red), among others. These steps would be repeated between module_*n*_ and module_*n* + 1_ till the termination module. Termination module of NRPS also contains an offloading domain such as a TE domain or reductive (R) domain after T domain to release the T-bound polypeptide intermediate for the completion of NRPS biosynthesis. The TE domain hydrolyzes or cyclize the intermediate to form a linear or cyclic polypeptide. Additionally, terminal C_*T*_ domain in fungal NRPSs disconnects the oligopeptide by macrocyclization, and R^∗^ domain mediates Dieckmann-type cyclization of PK-NRP hybrids to obtain tetramate moieties (Sussmuth and Mainz, 2017). Diversity of NRPS is largely present in the type II NRPSs that consist of standalone or minimal domains, which have been reviewed in detail in other papers (Jaremko et al., 2019; McErlean et al., 2019).

Due to the collinear and modular biosynthetic logic, the structural diversity of the products of modular PKS and NRPS could be largely attributed to a few variables (Kornfuehrer and Eustáquio, 2019). The modules of PKS and NRPS are suitable devices for retro-biosynthesis, which starts from the target product and proceeds backward to precursors by stepwise combination of the independent module reactions (Pang et al., 2019).

## Modular PKS and NRPS Engineering

**FIGURE 3:**
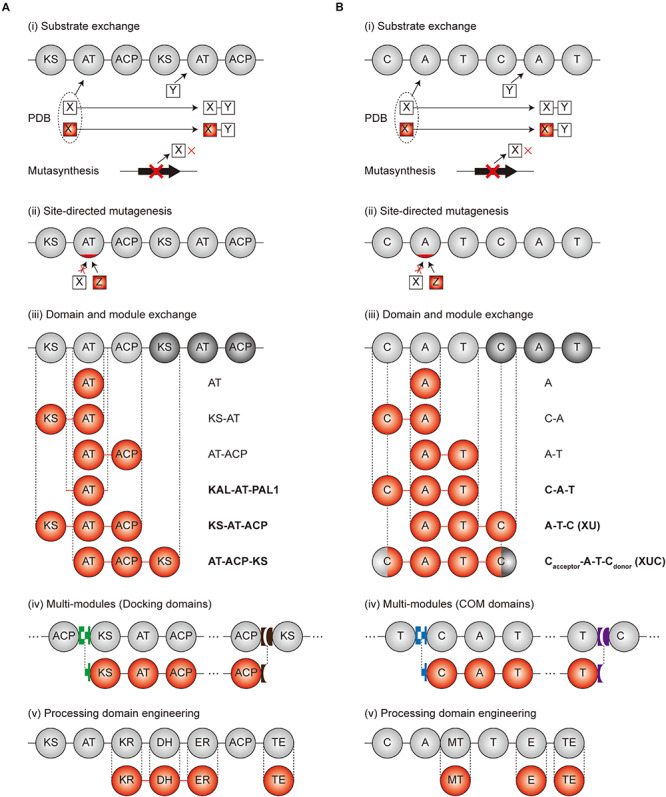
Engineering scheme for modular PKS and NRPS. **(A)** Engineering strategies of PKSs. **(B)** Engineering strategies of NRPSs. Gray circles and red circles indicate the original and modified domains, respectively. Green, brown, blue, and purple blocks, shaped as lock-and-key models, are the docking domains for **(A)** and COM domains for **(B)**, respectively. Linkers were indicated as the lines between the domains. In case of (iii) domain and module exchange, the exchangeable units are indicated at the right of the domains. The units indicated as bold characters are currently the best exchangeable units. PDB, precursor-directed biosynthesis.

As various strategies have been employed for the engineering of modular PKS and NRPS (Brown et al., 2018; Klaus and Grininger, 2018), we provided several landmark examples (Figure 3).

### Substrate Exchange

Early attempts for the engineering of PKSs and NRPSs were conducted via precursor directed biosynthesis (PDB) and mutasynthesis (Alanjary et al., 2019) (Table 1). PDB is a strategy of creating a new product by providing an external substrate instead of the native substrate of the recognition domain (AT and A domain). This strategy is based on the native promiscuity of the recognition domains that are able to incorporate more than one kind of substrate. The advantages of this strategy are that the native enzyme could be free of any genetic engineering, and understanding of the structures and mechanisms of the enzymes is not required. This is because the promiscuity is easily confirmed by adding the target substrates and then measuring the kinetic profiles or the product formation. Despite this, native substrate and alternative substrates compete for the enzyme reaction that results in the formation of a mixture of major and minor products, hampering the yield and purity of the desired product. One of the successful examples of PDB was the incorporation of a series of 21 substrates consisting of monocyclic, polycyclic, branched aliphatic acids, benzoic acids, and heterocyclic acids to AT domain in loading module of rapamycin PKS, suggested by the previous report of relaxed substrate specificity of the domain (Lowden et al., 2004). Among the novel rapamycin analogs, one monocyclic aliphatic acid product has immunosuppressant activity comparable to the native product. In another example, native substrate L-Pro was altered by non-proteinogenic amino acids for pyreudione NRPS to generate various derivatives from pyreudione E to pyreudione K (Klapper et al., 2018). Although there were more examples for successful PDB, most of their products showed lower yield compared to the native products (Ward et al., 2007; Moran et al., 2009; Crawford et al., 2011; Xie et al., 2014).

**TABLE 1:** Selected examples of the substrate exchange strategy.

**Category**	**Target**	**Engineering strategy (substrate change/genetic manipulation)**	**Product**	**References**
Precursor directed biosynthesis	6-deoxyerythronolide B PKS	Native precursor propyl-CoA change to diketide precursor (SNPCs)/DEBS LM deletion	15-fluoroethyl-6 deoxyerythronolide B	Ward et al., 2007
Precursor directed biosynthesis	Rapamycin PKS	Native precursor 4,5-dihydroxycyclohex-1-enecarboxylic acid change to 21 analogs	Monocyclic aliphatic acids	Lowden et al., 2004
Precursor directed biosynthesis	Pyreudione NRPS	Native precursor L-Pro change to prolin derivatives	Pyreudione E to K	Klapper et al., 2018
Precursor directed biosynthesis	Iturin A NRPS	Native precursor L-Tyr change to 3-fluoro-L-Tyr	Fluorinated iturin A	Moran et al., 2009
Mutasynthesis	Geldanamycin PKS	Native precursor 3-amino-5-hydroxybenzoic acid change to 18 analogs/3-amino-5-hydroxybenzoic acid (AHBA) biosynthetic gene deletion	Geldanamycin variants	Eichner et al., 2009
Mutasynthesis	DEBS1-soraphen hybrid PKS	Native precursor benzoate change to *p*-fluoro, *p*-hydroxy, *m*-hydroxybenzoate/*badA* deletion with *encN* insertion	Triketide lactone variants	Garcia-Bernardo et al., 2004
Mutasynthesis	FK506 PKS	Native precursor allylmalonyl CoA change to 4-methylpentanoic acid/*tcsB* deletion	36-methyl-FK506	Mo et al., 2011
Mutasynthesis	Balhimycin NRPS	Native precursor β-OH-Tyr change to 3-Fht/*bhp* deletion	Fluorobalhimycin	Weist et al., 2002
Mutasynthesis	Balhimycin NRPS	Native precursor dihydroxyphenylglycine change to hydroxylated or methoxylated phenylglycines/*dpgA* deletion	Hydroxylated or methoxylated balhimycin	Weist et al., 2004
Mutasynthesis	Salinosporamide NRPS	Native precursor 5′-CIDA change to 5′-FDA/*salL* deletion	Fluorosalinosporamide	Eustaquio and Moore, 2008

To overcome the competition between native and altered substrates, mutasynthesis approaches were considered to increase the yield and purity of the desired product by feeding the alternative substrates together with the deletion of the biosynthetic genes of the native substrates in the expression host. For example, the mutasynthesis of novel FK506 derivatives was reported by the deletion of *tcsB*, resulting in modification of the FK506 C21 moiety (Mo et al., 2011). Utilizing the native promiscuity of AT domain of module 4 in FK506 PKS, the feeding of *trans*-2-hexenoic acid, 4-methylpentanoic acid, and 4-fluorocrotonic acid generated 36,37-dihydro-37-methyl-FK506, 36- methyl-FK506, and 36-fluoro-FK520, respectively. Interestingly, 36-methyl-FK506 not only has immunosuppressant function but has also improved neurite outgrowth activity. Other examples obtained various derivatives through incorporation of the non-natural units by mutasynthesis (Weist et al., 2002, 2004; Garcia-Bernardo et al., 2004; Eustaquio and Moore, 2008; Eichner et al., 2009). However, the requirement of genetic engineering and the dependency of native promiscuity are limitations of mutasynthesis. Despite these limitations, mutasynthesis is still useful when applied together with domain and module engineering in a synergistic manner. For example, non-natural precursors generated by semisynthetic or click chemistry could be fed, accompanied by the deletion of the native precursor biosynthetic genes and the mutations of PKS or NRPS enzymes to attach the pharmacophore-containing moiety or dye for novel chemical production (Alanjary et al., 2019).

### Mutagenesis of Substrate Recognition Domains

The AT domain of PKS or the A domain of the NRPS have been major mutation targets for enzyme engineering (Table 2). Two directions of mutagenesis have been applied; (i) reducing or increasing the native promiscuity of the domain and (ii) creating *de novo* specificity of the domain (Musiol-Kroll and Wohlleben, 2018; Stanisic and Kries, 2019). Compared to domain and module exchange (see subsection “Domain and Module Exchange”), the modification of several residues in the AT or A domain could minimize the structural perturbation of entire enzyme assemblies, as well as minimally affecting the protein–protein interactions between adjacent domains and modules (Brown et al., 2018). In addition, this approach fundamentally changes the protein-substrate interaction enabling the incorporation of non-natural synthetic substrates.

**TABLE 2:** Selected examples of the substrate recognition domain mutagenesis.

**Target**	**Engineering strategy (substrate change (mutation))**	**Rationale for engineering**	**Product**	**References**
AT domain in module 4 of DEBS PKS	Methylmalonyl-CoA (YASH) to malonyl-CoA (HAFH)	Domain sequence alignment and structure-based selection	6-desmethyl-6-dEB	Reeves et al., 2001
AT domain in module 3 of epothilone PKS	Both methylmalonyl, malonyl-CoA (HASH) to methylmalonyl-CoA (YASH) or malonyl-CoA (HAFH)	Domain sequence alignment	Triketide lactones derivatives	Petkovic et al., 2008
AT domain in module 2 of DEBS PKS	Methylmalonyl-CoA (YASH) to non-natural propargyl, ethyl, allylmalonyl (VASH)	Domain sequence alignment and structure-based selection	Triketide lactones derivatives	Vogeli et al., 2018
AT domain in module 6 of DEBS PKS	Methylmalonyl-CoA to diverse bulky extender units (Q,Y,S to A,G,R)	Structure-based selection	Propargyl, heptenyl, benzyl-SNAC incorporation	Li et al., 2018
AT domain in module 5,6 of pikromycin PKS	Methylmalonyl-CoA to propargyl, ethyl, allyl, butylmalonyl CoA (Y755V, Y753V)	Domain sequence alignment and Homology modeling	10-dML analogs, narbonolide analogs	Kalkreuter et al., 2019
AT domain in module 1 of avermectin PKS	40 carboxylic acids to (2S)-methylbutyric acid (V222L or V222A)	Homology modeling	(2S)-methylbutyric acid (isolated module)	Wang et al., 2015
AT domain in module 6 of DEBS PKS	Methylmalonyl-CoA (YASH) to non-natural alkynyl-modified extender unit (RASH)	Domain sequence alignment and saturation mutagenesis	10-dML analogs	Koryakina et al., 2017
A domain of module 2 of surfactin NRPS	L-Asp to L-Asn (V299I, H322E, I330V)	Domain sequence alignment and Homology modeling	[Asn5]surfactin	Eppelmann et al., 2002
A domain of module 10 of CDA NRPS	L-Glu, Me-Glu to L-Gln, Me-Gln (K278G, Q236G)	Domain sequence alignment	CDA4a-10mQ	Thirlway et al., 2012
A domain of module 3 of Fusaricidins NRPS	L-Tyr, L-Val, L-Ile, L-(allo)-Ile, or L-Phe to only L-Phe (three mutants of three sites)	Domain sequence alignment	Fusaricidin analog (LI-F07)	Han et al., 2012
A domain of module 1 of Anabaenopeptin NRPS	L-Arg, L-Tyr to 4-azido-Phe (E204G,S243E or S243H)	Structure-based selection	Novel anabaenopeptin analog, clickable	Kaljunen et al., 2015
A domain of module 1 of gramicidin S NRPS	L-Phe to L-Tyr, *O*-propargyl-L-Tyr (W239S)	Structure-based selection and saturation mutagenesis	Propargylated DKP, clickable	Kries et al., 2014
A domain of module 1 of syringomycin NRPS	A domain of L-Ser-specifying EntF exchange to X-specifying SyrE. None to L-Ser (3.2 amino acid change per clone)	Domain sequence alignment, directed evolution (2 rounds), and iron growth selection	Enterobactin derivatives	Fischbach et al., 2007
A domain of AdmK of andrimid NRPS/PKS hybrid	L-Val to L-Ile, L-Leu, L-Ala, L-Phe (three sites saturation mutagenesis)	Domain sequence alignment, directed evolution, and high-throughput LC-MS/MS	Andrimid derivatives	Evans et al., 2011
A domain of module 1 of tyrocidine NRPS	L-Phe to L-Thr (A301C, C331I, I3330V, W239M)	Domain sequence alignment, directed evolution, and PPi exchange assay	Tyrocidine derivatives	Villiers and Hollfelder, 2011
A domain of module 1 of bacillibactin NRPS	2,3-dihydroxybenzoic acid to 3-hydroxybenzoic acid and 2-aminobenzoic acid (four sites mutations)	Domain sequence alignment, directed evolution, and yeast surface display with FACS	3-hydroxybenzoic acid and 2-aminobenzoic acid (isolated module)	Zhang et al., 2013

The substrate specificity of the AT domain is determined by approximately 100 residues toward the C-terminus from the active site serine (Musiol-Kroll and Wohlleben, 2018). The most common substrates of elongation AT domains are malonyl- or methylmalonyl CoA (Chan et al., 2009; Barajas et al., 2017), while at least 20 malonyl-CoA analogs have been found to be incorporated (Yuzawa et al., 2017b). The substrates of the loading AT domain are more variable (Barajas et al., 2017). Sequence alignment analysis of prototypical 6-deoxyerythronolide B synthase (DEBS) and epothilone PKS suggested the specificity code is ‘HAFH’ for malonyl-CoA, ‘YASH’ for methylmalonyl-CoA, and ‘HASH’ for the both (Reeves et al., 2001; Petkovic et al., 2008). Changing the code results in altered specificity to corresponding substrates, although there may be the mixed substrate specificity for both substrates. The specific residues for changing the promiscuous specificity toward non-natural extender units were determined based on the domain sequence alignment, X-ray crystal structure, and homology modeling (Reeves et al., 2001; Petkovic et al., 2008; Li et al., 2018; Vogeli et al., 2018; Kalkreuter et al., 2019).

The specificity conferring code of the A domain in NRPS is more variable than PKS. Early studies about X-ray structure and sequence alignment suggested the eight specificity codes, referred to as Stachelhaus codes, which were later updated to broader range of residues by computational modeling (Conti et al., 1997; Stachelhaus et al., 1999; Challis et al., 2000; Rottig et al., 2011). Based on this, the A domain in the second module of surfactin NRPS was mutated at three of the eight specificity conferring residues (V299I, H322Q, and I330V), resulting in the successful L-Asp to L-Asn specificity change (Eppelmann et al., 2002). Moreover, the corresponding code of A domain in the third module of fusaricidins NRPS was determined by aligning its residues to those of the L-Phe specific A domain of gramicidin S and tyrocidine S NRPS. These mutations resulted in increased specificity toward L-Phe to produce the fusaricidin analog (LI-F07) (Han et al., 2012). Other examples are presented in Table 2.

The limitations of the site-directed mutagenesis approach are the effects of the other residues, outside the specificity conferring code. For instance, the specialized protein–protein interactions for the different PKSs and NRPSs such as KS-AT and C-A interfaces impeded the establishment of the universal code and the accurate prediction of specificity. This is also consistent with the frequent results of the unexpected mixed substrate specificity by AT or A domain mutagenesis. To overcome this, the directed evolution method that identifies the desired clone among the random mutagenesis libraries of 10^4^ to 10^6^ clones by iterative selection cycles and high-throughput screening has been reported. The saturation mutagenesis of three sites in the AT domain of andrimid PKS generated a library of approximately 14,000 mutants, which were analyzed via the highly sensitive LC-MS/MS screening method (Evans et al., 2011). Consequently, mixed derivatives of adrimid containing L-Ile, L-Leu, L-Ala, and L-Phe, modified from L-Val, respectively, were obtained with improved antimicrobial activity. Together with other examples, the directed evolution approach was expected to become the universal strategy for the specificity change regardless of the kind of the enzymes (Fischbach et al., 2007; Villiers and Hollfelder, 2011; Zhang et al., 2013). However, there are still several requirements for feasible directed evolutions such as high-throughput assays, iterative cycles of mutagenesis for stability and selectivity, and effective screening. Alternatively, reducing the library size by the prediction of the residues for specificity change based on structural information and evolutionary evidence would be developed.

### Domain and Module Exchange

Parallel to the site-directed mutagenesis approach, the domain or module exchange approaches have been applied to change the substrate specificity of PKS and NRPS (Sussmuth and Mainz, 2017; Kornfuehrer and Eustáquio, 2019). As the exchanging domains and modules are generally well-studied, the substrate specificity change is predictable and experimental design is more convenient. However, the correct splicing site should be precisely determined to preserve the protein–protein interactions between the acceptor and donor units, to minimize the perturbation of sophisticated conformational changes during chain elongation, and to maintain the overall structure of PKS and NRPS.

#### Domain or Module Exchange of PKS

In the case of PKS, successful examples were observed for the AT domain of the well-studied DEBS PKS exchange to other AT domains in different modules of DEBS PKS, or the modules from other PKSs (Oliynyk et al., 1996; Stassi et al., 1998; Hans et al., 2003; Petkovic et al., 2003) (Table 3). This AT exchange strategy is normally referred as ‘AT domain swapping.’ The splicing sites were determined as the rough boundaries of AT domains inferred by the sequence alignment between similar modules, and the AT domains were cloned by providing synonymous mutations to the boundaries for the introduction of restriction enzyme sites (Patel et al., 2004). Nevertheless, the swapping of only the AT domain was unfavorable in terms of impairment of catalytic activity owing to the non-native interactions between the swapped domains and neighboring domains such as KS, ACP, and interdomain linkers. The X-ray structural studies and mutagenesis studies of KS-AT and AT-ACP didomains revealed crucial residues in the interdomain linkers and domain interfaces for the specificity or catalytic activity, suggesting that the KS or ACP domain should be exchanged together with the AT domain (Kuhstoss et al., 1996; Marsden et al., 1998; Kim et al., 2004; Chen et al., 2007; Yuzawa et al., 2012; Miyanaga et al., 2016; Murphy et al., 2016). Recently, sequence alignments and structural studies related to module 6 of DEBS PKS and module 1 of β-lipomycin PKS provided ideal splicing sites located at interdomain linkers adjacent to AT domain despite of the protein–protein interactions with KS and ACP domains (Yuzawa et al., 2017b). The splicing sites were located at the conserved GTNAHVILE region of KS-AT linker (KAL) and conserved LPTY(A/P)FQ (H/R)xRYWL region of post AT linker 2 (PAL2) to minimize the non-native adjacent sequences, resulting in the universal KAL-AT-PAL1 unit for AT domain swapping. However, swapping by the KAL-AT-PAL1 unit is still remained to be validated for more various PKSs, because it may alter the protein–protein interactions between the domains and disrupt gatekeeping from downstream processing, which may result in incompatibility (Barajas et al., 2017).

**TABLE 3:** Selected examples of domain or module exchange of modular PKS.

**Exchange unit**	**Target**	**Engineering strategy (substrate change/donor unit/fusion point)**	**Rationale for engineering**	**Product**	**References**
AT-PAL1	AT domain in module 1 of DEBS PKS	Methylmalonyl to malonyl CoA specific/rapamycin AT domain in module 2 exchange/N,C-term RE site splicing	Domain sequence alignment	Two novel triketide lactones	Oliynyk et al., 1996
AT-PAL1-PAL2	AT domain in module 6 of DEBS PKS	Methylmalonyl to methylmalonyl or malonyl CoA specific/RAPS AT2, DEBS AT4, DEBS AT5 exchange/interdomain region RE site splicing	Domain sequence alignment	Triketide lactones derivatives	Hans et al., 2003
AT-PAL1	AT domain in module 4 of DEBS PKS	Methylmalonyl to malonyl CoA specific/rapamycin AT domain in module 2 exchange/N,C-term RE site splicing	Domain sequence alignment	6-desmethyl erythromycin D	Petkovic et al., 2003
AT-PAL1	AT domain in module 4 of DEBS PKS	Methylmalonyl to ethylmalonyl CoA specific/niddamycin AT domain in module 5 exchange/N,C-term RE site splicing	Domain sequence alignment	6-desmethyl-6-ethylerythromycin A	Stassi et al., 1998
AT-PAL1-PAL2	AT domain in module 1-5,7 of geldanamycin PKS	Methylmalonyl or methoxymalonyl to malonyl CoA specific/rapamycin AT domain in module 2,14 exchange/N,C-term RE site splicing	Domain sequence alignment	Geldanamycin derivatives	Patel et al., 2004
AT-ACP	LM (AT-ACP) of DEBS PKS	Methylmalonyl or malonyl CoA to 40 carboxylic acids specific/AVES LM (AT-ACP) exchange/ACP C-term region splicing	Domain sequence alignment	Novel antibiotic erythromycins	Marsden et al., 1998
KS^*Q*^-AT-ACP	LM (KS^*Q*^-AT-ACP) of tylactone PKS	Malonyl to Methylmalonyl CoA specific/Platenolide LM (KS^*Q*^-AT-ACP) exchange/ACP-KS RE site splicing, synthetic linker connect	Domain sequence alignment	16-methyl platenollde I	Kuhstoss et al., 1996
KAL-AT-PAL1	AT domain in module 6 of DEBS PKS, AT domain in module 1 of β-lipomycin PKS	Methylmalonyl to methylmalonyl or malonyl CoA specific/epothilone AT domain in module 4 exchange, other various AT domains exchange/KAL-AT-PAL1	Domain sequence alignment and structure-based selection	3-hydroxycarboxylic Acid, short-chain ketones	Yuzawa et al., 2017b
KS-AT-KR-ACP	Insertion between module 1,2 (KS-AT-KR-ACP) of DEBS PKS	Methylmalonyl, malonyl CoA specific added/rapamycin module 2,5 (KS-AT-KR-ACP, AT-KR-ACP-KS) insertion/KS N-term, C-term RE site splicing	Domain sequence alignment	Novel octaketide macrolactones	Rowe et al., 2001
KS-AT-ACP, multi-modules	Modules of DEBS PKS	Same specificity/M1-M3, M1-M6, M1-RifM5, M2 to RifM5/Native RE site, conserved region of interpolypeptide linker	Domain sequence alignment	Triketide lactones derivatives	Gokhale et al., 1999
KS-AT-ACP	Modules of DEBS, soraphen, epothilone, geldanamycin, rifamycin, rapamycin, pikromycin, leptomycin PKS	Two module combinatorial biosynthesis of 14 module of 8 PKS/Conserved region of KS N-term and ACP C-term RE spicing	Domain sequence alignment	Triketide lactones derivatives	Menzella et al., 2005
KS-AT-ACP	Modules of DEBS 1,2,3 PKS	Methylmalonyl to propionyl, methylmalonyl, malonyl CoA specific/3 module combinatorial biosynthesis/conserved region of interpolypeptide linker	Domain sequence alignment	Triketide lactones derivatives	Klaus et al., 2016
AT-DH-KR-ACP-KS	Module 2 of neoaureothin PKS	Deletion of methylmalonyl CoA specific module 2/KS-AT linker conserved region and docking domain of ACP RE splicing	Domain sequence alignment	Homoaureothin	Sugimoto et al., 2015
KS-AT-ACP, KS-AT	Module 6 of DEBS PKS	Methylmalonyl-CoA to methylmalonyl or malonyl CoA specific/module 2,3,5 of DEBS PKS exchange/docking domain exchange to SYNZIP	Domain sequence alignment and structure-based selection	Triketide lactones derivatives	Klaus et al., 2019

In addition to determination of AT domain splicing sites, the exchange of a whole module (KS-AT-ACP) could be the alternative strategy to change the specificity. In this case, the interactions between upstream ACP and downstream KS domain should be considered, including covalent interdomain linkers and non-covalent interactions between domains. By splicing at the appropriate site of the interdomain linkers, 14 KS-AT-ACP modules from 8 PKSs was successfully isolated as a functional unit, and connected to generate a total of 154 combinatorial bimodular PKSs (Gokhale et al., 1999; Menzella et al., 2005). On the other hand, recent studies suggested that the evolutionary functional module is AT-ACP-KS rather than conventional KS-AT-ACP (Sugimoto et al., 2015; Keatinge-Clay, 2017). The sequence alignment of four aminopolyol PKSs supported this unit that the higher evolutionary correlation of the sequences between processing domains and downstream KS domain compared to the upstream KS domain (Zhang et al., 2017). Also, the first half of post-AT linker sequence showed higher correlation to the AT domain than the KS-AT linker, which refers AT-(processing domains)-ACP-KS is a more evolutionarily conserved unit. Moreover, the multi-modules of PKSs with the interpolypeptide non-covalent docking domain (DD) at both ends were able to be exchanged. The DD pairs were the compatible parts for exchanging the subunits that, using heterologous DD pairs led to the successful production of diketides and triketides (Menzella et al., 2005). However, several examples showed severe impairment of catalytic activity (Klaus et al., 2016). This is because the chain translocation step from the ACP domain of upstream module to the KS domain of downstream module was the bottleneck in addition to the chain elongation step (Khosla et al., 2007; Klaus et al., 2016), emphasizing the importance of ACP-KS interaction, even when the DDs are compatible. Besides exchanging the module, incorporating the growing polypeptide with similar chain length to the native module was more successful than those of different chain length. Overall, the modular exchange strategies for PKS are diverse but the protein–protein interactions particularly for ACP-KS domains are important.

#### Domain or Module Exchange of NRPS

Similar to the exchange of domains and modules in PKSs, many engineering attempts for NRPSs have been reported. The swapping of only an A domain successfully resulted in the alteration of the specificity by the determination of proper splicing sites but, they were often hindered by non-native interactions between the swapping domains and neighboring domains such as C, T, and interdomain linkers (Stachelhaus et al., 1995; Doekel and Marahiel, 2000) (Table 4). The exchange of C-A didomain was more successful than the single A domain exchange in surfactin and pyoverdine NRPS (Tanovic et al., 2008; Calcott et al., 2014) but, failed in another report (Ackerley and Lamont, 2004). A-T didomain had a short interdomain linker with a conserved LPxP motif to interact with a key L-Tyr residue in the C-terminus of the A domain, and to assist the movement of the T domain during the catalytic cycle (Beer et al., 2014; Miller et al., 2014). Based on these characteristics, the A-T didomain in module 2 of actinomycin NRPS was successfully exchanged to A-T didomain in module 5 of actinomycin NRPS, but it still had low a yield (Schauwecker et al., 2000).

**TABLE 4:** Selected examples of domain or module exchange of modular NRPS.

**Exchange unit**	**Target**	**Engineering strategy (substrate change/donor unit/fusion point)**	**Rationale for engineering**	**Product**	**References**
A	A domain in module 7 of surfactin NRPS	L-Leu to L-Phe, L-Orn, L-Cys, L-Val specific/gramicidin S A domain in module 1,4,5 and ACV A domain in module 2,3 exchange/N,C-term RE site splicing	Domain sequence alignment	Five different surfactin variants	Stachelhaus et al., 1995
A	A domain in module 3 of hormaomycin NRPS	(β-Me)Phe to L-Thr, (3-Ncp)Ala, L-Val specific/Hormaomycin A subdomain in module 2,4,6 exchange/N,C-term RE site splicing	Homology modeling	Altered substrates (A domain assay)	Crüsemann et al., 2013
A, T-C-A	A domain in module 8,9 of tyrocidine NRPS	L-Orn and L-Leu to L-Ile, L-Phe specific/A domain in module 1 of bacitracin A NRPS/tyrocidine A (or T-C-A) domain in module 3/RE site splicing and fusion to TCALeuTTe.	Domain sequence alignment	Dipeptides (isolated module)	Doekel and Marahiel, 2000
A, C-A	A domain in module 10 of pyoverdine NRPS	L-Thr to L-Thr, L-Lys, L-Ser specific/Pyoverdine A domain in module 8, pyoverdine A domain in 3,4,5 modules, (A or C-A) of NRPS of other species exchange/deletion and genome integration by *attB*.	Domain sequence alignment	Pyoverdine derivatives	Calcott et al., 2014
A-T	A domain in module 2 of actinomycin NRPS	L-Val to *N*-methyl valine specific. Actinomycin (A-T) domain in module 5 exchange/C-A linker region, post T site RE splicing	Domain sequence alignment	Acyl-threonine–MeVal (isolated module)	Schauwecker et al., 2000
C-A-T, C-A-T-E	Modules 8-13 of daptomycin NRPS	L-Ala, L-Ser, 3 mGlu, L-Kyn to (L-Ser, L-Lys, L-Asn), (L-Ala, L-Asn), L-Glu, L-Trp, L-Ile specific/Modules 8-11 of A54145 NRPS/T-C, T-E, E-C linker RE splicing	Domain sequence alignment	Daptomycin derivatives	Nguyen et al., 2006
C-A-T, C-A-T-E	Modules 2,3,8,11,12,13 of A54145 NRPS	L-Glu, L-Asn, L-Lys, L-Asn, 3 mGlu to L-Asn, L-Asp, (L-Ala, L-Ser, L-Asn), (L-Ala, L-Ser), L-Glu specific/Many modules of A54145 NRPS/T-C, T-E, E-C linker RE splicing	Domain sequence alignment	A54145 derivatives	Nguyen et al., 2010
C-A-T-C	Modules 1,5 of surfactin NRPS	L-Glu, L-Asp to L-Gln, L-Asp specific/Module 1,5 of lichenysin A NRPS/C domain (HHXXXDG) active-site splicing	Domain sequence alignment	Recombinant lipopeptides	Yakimov et al., 2000
C-A + T-E	Insertion between module 4 and 5 of balhimycin NRPS	L-Hpg specific added/C_5_A_5_T_4_E_4_ of balhimycin NRPS/A-T linker region splicing	Domain sequence alignment	Balhimycin derivatives	Butz et al., 2008
A-T-C	Modules of xenotetrapeptide NRPS	Various changes/recombination of XtpS, GxpS, KolS, AmbS, GarS, GrsB, BicA/C-A linker region (WNATE) splicing.	Domain sequence alignment and structure-based selection	Xenotetrapeptides	Bozhuyuk et al., 2018
C_*acceptor*_-A-T-C_*donor*_	Modules of xenotetrapeptide NRPS	Various changes/recombination of XtpS, GxpS, KolS, AmbS, GarS, GrsB, BicA, SrfA, GrsAB, TycC, XeyS, Pax, others/C_*acceptor*_-C_*donor*_ linker splicing.	Domain sequence alignment and structure-based selection	Xenotetrapeptides	Bozhuyuk et al., 2019
C-A-T-E, multi-modules	Modules 4,5,6 of surfactin NRPS	L-Val, L-Asp, D-Leu to none/multimodule skipping by docking domain change/T/E-COM_*D*_ or COM_*A*_-C linker splicing	Domain sequence alignment	Surfactin derivatives	Chiocchini et al., 2006

Considering both interactions within the C-A and A-T didomains, C-A-T module exchange occurred in several NRPSs. The most representative examples were, the daptomycin and A54145 NRPS modules of C-A-T or C-A-T-E spliced at the interdomain linker of the T and C domain that were changed to produce the novel daptomycin and A54145 derivatives (Yakimov et al., 2000; Nguyen et al., 2006, 2010; Butz et al., 2008). But the protein–protein interactions between T and C domain, consisting of a variable interdomain linker, were disturbed resulting in impaired activity. Different from the transacylation of PKS which leads to two separated steps of upstream ACP to KS, and then KS to downstream ACP, the C domain governs the peptide bond formation by one step and has strong stereoselectivity and side-chain selectivity for both donor and acceptor peptides (Clugston et al., 2003; Samel et al., 2007; Bloudoff and Schmeing, 2017; Li et al., 2017). Due to this strong correlation between all adjacent domains and linkers, finding the optimal splicing sites for the module exchange has remained a bottleneck. Recently, a suggested exchangeable unit A-T-C (XU) that the C-A linker was thought to be a better splicing site than other linkers in view of the N-terminal conserved region, the absence of secondary structures, and the fewer interactions between other domains (Bozhuyuk et al., 2018). Nonetheless, there was a requirement for the exchange of A-T-C unit that the C domain has specificity filters for both upstream and its own module. Therefore, downstream A-T-C unit should also be exchanged together with the target A-T-C unit. To avoid the limitations of XU, the XUC exchange unit which is C_*acceptor*_-A-T-C_*donor*_ with the splicing site located at the intradomain conserved linker was newly suggested (Bozhuyuk et al., 2019). Through dissecting the C domain specificity for upstream and downstream module, it was theoretically ideal that the exchange unit could be fused in a combinatorial manner. However, experimental results showed that the fusion between units from different genera caused a decrease in the yield. As the overall structure disruption by the C domain was thought to be the main reason, the solution to complement this limitation would be to research and discover more exchangeable XUC units.

There were several successful multi-module exchanges produced by using communication (COM) domain pairs such as DD domain pairs of PKS (Hahn and Stachelhaus, 2004; Chiocchini et al., 2006). In addition, the specificity of COM domain pairs has the ability to be altered via modification at their key residues resulting in the non-native COM domain (Hahn and Stachelhaus, 2006). Some examples, however, displayed a low product yield due to the disruption of non-covalent protein–protein interactions between the T and C domain. Overall, the modular exchange strategies for NRPS are diverse, similar to PKS, but the protein–protein interactions between domains, particularly those involving the C domain, are commonly important considerations.

Currently, the favorable strategies of exchanging module specificity for the production of novel chemical derivatives seems to be; (i) KAL-AT-PAL1 unit, AT-ACP-KS unit, using heterologous DD pairs for PKS, and (ii) XU, XUC, using heterologous COM domain pairs for NRPS, respectively. Although numerous studies for altering specificity of PKS and NRPS have been reported, more engineering trials should be accumulated for various PKSs and NRPSs to optimize the strategies employed.

### Processing and Offloading Domain Engineering

In addition to the module specificity change, the incorporated extender unit could be further modified by various processing domains. Most of the processing target residues were the α-substituent and β-keto group of acyl-ACP intermediate of PKS, and R group of amino acid intermediate of NRPS (Barajas et al., 2017; Sussmuth and Mainz, 2017) (Table 5).

**TABLE 5:** Selected examples of processing and offloading domain engineering.

**Target**	**Engineering strategy [engineering (+donor)/target sites]**	**Rationale for engineering**	**Product**	**References**
KR domain in module 6 of DEBS PKS	KR inactivation/Y159F, S146A, K163E mutation	Domain sequence alignment and Homology modeling	3-Keto derivative of 6-deoxyerythronolide B	Reid et al., 2003
KR domain in module 2 of DEBS PKS	A1-type KR change to eight A2-type KR domains (amphotericin KR1, KR11, concanamycin KR4, KR10, elaiophylin KR4, oligomycin KR5, pimaricin KR7, candicidin KR13) and six B2-type KRs (DEBS KR1, lankamycin KR1, pikromycin KR1, lasalocid KR7, ECO-02301 KR19, stambomycin KR21)/AT-KR linker and KR-ACP linker RE splicing	Domain sequence alignment	Epimerized triketide lactones	Annaval et al., 2015
KR domain in module 1 of lipomycin PKS	A2-type KR change to A1-type amphotericin KR2 (or +DE2), and B1-type concanamycin KR2 (or +DE2)/post AT linker before DE and KR-ACP linker RE splicing	Domain sequence alignment and structure-based selection	*syn* form hydroxyacids	Eng et al., 2016
KR domain in module 2 of DEBS PKS	A1-type KR change to A2-type amphotericin KR2 and amphotericin KR11 with cognate DE/post AT linker before DE and KR-ACP linker RE splicing	Domain sequence alignment and structure-based selection	2D,2L-triketide lactone, ketolactones	Zheng et al., 2013
DH domain in module 18 and KR domain in module 21 of FR-008 PKS	KR and DH inactivation/Y1526F for KR21, H3084Y for DH18.	Domain sequence alignment	FR-008-V, -III, and -VI	Zhou et al., 2008
ER domain in module 4 of DEBS PKS	ER stereochemistry altered/Y52V for ER4 domain.	Domain sequence alignment and homology modeling	Triketide lactone with S to R methyl branch configuration switched	Kwan et al., 2008
KR domain in module 1 of borrelidin PKS	BorKR1 to Reductive loop of SpnB of spinosyn PKS and SpnBDH1 (in *cis*-double bond) to BorDH2 (in *trans*-double bond)/AT-DH linker region and ER-ACP linker region splicing	Domain sequence alignment, structure-based selection and Ppant ejection	Adipic acid	Hagen et al., 2016
TE domain in module 9 of tautomycetin PKS	Linear release change to macrocyclization by using TE domain in module 6 of pikromycin/TMC TE linker downstream splicing	Domain sequence alignment	Cyclized tautomycetin analog	Tripathi et al., 2016
E domain in module 4 of tyrocidine NRPS	L-Phe-D-Phe-L-Pro change to D-Phe-L-Pro. TycB2-3-AT.CATE(E_*TycA*_ or E_*TycB*_) + TycB1-CAT/Te/T-E linker splicing	Domain sequence alignment	D-Phe-L-Pro (isolated module)	Stein et al., 2006
MT domain in module 2 of bassianolide NRPS, module 2 of beauvericin NRPS	*N*-methyl-L-Leu, *N*-methyl-L-Phe change to L-Leu, L-Phe. MT deletion/Intact domain deletion by overlap extension (SOE) PCR	Domain sequence information	*N*-desmethylbassianolide, *N*-desmethylbeauvericin B	Xu et al., 2019
MT domain insertion to module 6 of echinomycin NRPS	L-Ser change to *O* or *N*-methylated Ser. *O*-methylating MT domain of module 4 of kutznerides NRPS and *N*-methylating MT domain of module 3 of thiocoraline NRPS/intact domain insertion by RE splicing	Domain sequence alignment	*O* or *N*-methylated Ser (isolated module)	Lundy et al., 2018
Tyrocidine derivative with D-Phe4 connected to PEGA resin	D-Phe4 change to various amino acid libraries. TycC TE isolated for macrocyclization/intact domain splicing	Domain sequence alignment, combinatorial solid-phase chemistry	Cyclized tyrocidine analogs	Kohli et al., 2002

#### Processing and Offloading Domain Engineering of PKS

In PKS, ketoreductase (KR), dehydratase (DH), and enoyl-reductase (ER) domains are the most abundant processing domains located between the AT and ACP domain as a reductive loop, which governs the degree of β-carbonyl reduction. The KR domain performs the NADPH-mediated reduction of β-keto groups to β-hydroxyl groups and determines the stereochemistry of α-substituent and β-hydroxyl group. As the full deletion of the KR domain showed loss of specificity due to impaired protein folding and stability, the inactivation of the KR domain by its key residue mutations was more effective (Reid et al., 2003). In another approach the stereochemistry was altered by KR domain swapping, resulting in the effective stereocontrol of the product, which was generally difficult through modern synthetic methods (Annaval et al., 2015; Eng et al., 2016). Notably, substrate specificity of the KR domain has been shown to be related to its substrate size, as it tends to be less active on smaller non-native substrates (Zheng et al., 2013). Next to the KR domain, DH domain promotes the dehydration of the β-hydroxyl group to form the double bond between α- and β-carbons, and even between β- and γ-carbons in some cases. Inactivation of DH domain by single mutation at the conserved active site motif resulted the retention of the β-hydroxyl group without specificity loss of the AT domain (Zhou et al., 2008). For the insertion of a non-native DH domain, it was important to consider the stereoselectivity of DH domain to the hydroxylated product by the upstream KR domain, which means that DH domain activity is strongly dependent to cognate KR domain (Barajas et al., 2017). Additionally, DH domain was also sensitive to the length of substrates that DH domains acting on natively long chain showed impaired activity to the shorter chain substrate (Faille et al., 2017). Lastly, ER domain promotes the reduction of the unsaturated α–β double bond formed by DH domain to generate the single bond, and determines the stereochemistry of the α-carbon. As the substrate of ER domain is the product of DH domain, the specificity of ER domain is dependent to both cognate KR and DH domain. Inactivation of ER domain by single mutation at the conserved residue switched the methyl branch configuration of triketide lactone product (Kwan et al., 2008). Moreover, ER domain affected the downstream ACP arrangement by the protein–protein interactions resulting in the different stereocontrol (Zhang et al., 2018). Three domains (KR, DH, ER) could be simultaneously introduced for the exchange of the KR domain in module 1 of borrelidin PKS (Hagen et al., 2016). An offloading TE domain was abundantly fused to other modules using the splicing site at the conserved linker between the offloading domain and ACP domain to produce truncated products. Moreover, they were regio-, stereospecific, which may alter the release and cyclization nature via swapping (Pinto et al., 2012; Barajas et al., 2015; Tripathi et al., 2016). Further engineering of other processing domains, such as the methyltransferase (MT) domain, should be conducted to utilize their potential to produce novel chemicals (Yuzawa et al., 2017c).

#### Processing and Offloading Domain Engineering of NRPS

Among a large variety of processing domains for non-ribosomal peptides, in this review we focused on the *cis*-acting processing domains located in the module.

Epimerase (E) domain governs *in situ* epimerization of the α-carbon of the T domain tethered L-amino acid during peptide elongation to generate D-amino acid. One example was the exchange of E domain in module 4 of tyrocidine NRPS (E_*Tyc*_B) to E domain in loading module of tyrocidine NRPS (E_*Tyc*_A), that showed the epimerization of L-Phe to D-Phe (Stein et al., 2006). Methyltransferse (MT) domain is another example of the engineering that provides *N*-methylation of the amino acid. This domain is usually integrated inside of A domain such as MT domain of cyclosporine A NRPS (Velkov et al., 2011). A recent engineering example of the MT domain in module 2 of bassianolide synthetase showed that the deletion of this domain generated the *N*-desmethylbassianolide without affecting the enzyme assembly lines (Xu et al., 2019). In another study, *O*-methylating MT domain was inserted between the A subdomains, resulting in the incorporation of *O* or *N*-methylated serine (Lundy et al., 2018). An offloading domain of NRPS was abundantly fused to other modules, similar to PKS, using the splicing site at the conserved linker between itself and the T domain to produce truncated products. Particularly, TE_*TycC*_ domain of tyrocidine NRPS was fused to other modules to generate various cyclized analogs by using its macrocyclization property (Kohli et al., 2001, 2002; Trauger et al., 2001).

Cyclization (Cy), Oxidase (Ox), and Formylation (F) domain were also expected to be the favorable targets for engineering. However, there have been no examples due to the upstream substrate specificity as well as other several mechanistic problems (Miller and Walsh, 2001; Schneider et al., 2003; Schoenafinger et al., 2006; Sussmuth and Mainz, 2017). Besides, other in-*trans* acting tailoring enzymes have the potential to be utilized for the production of novel derivatives but, studies are lacking. Therefore, further studies and engineering about various NRPS processing domains, including both in-*cis* and in-*trans* acting, should be quantitatively increased.

To sum up, the inactivation and swapping of the individual processing domain based on homology modeling and sequence alignment was successful, however, specialized protein–protein interactions hampered the full engineering of these domains, this required the case-by-case optimization of the swapping region.

## Repurposing Modular PKS and NRPS to Construct *de novo* Biosynthetic Pathways

**FIGURE 4:**
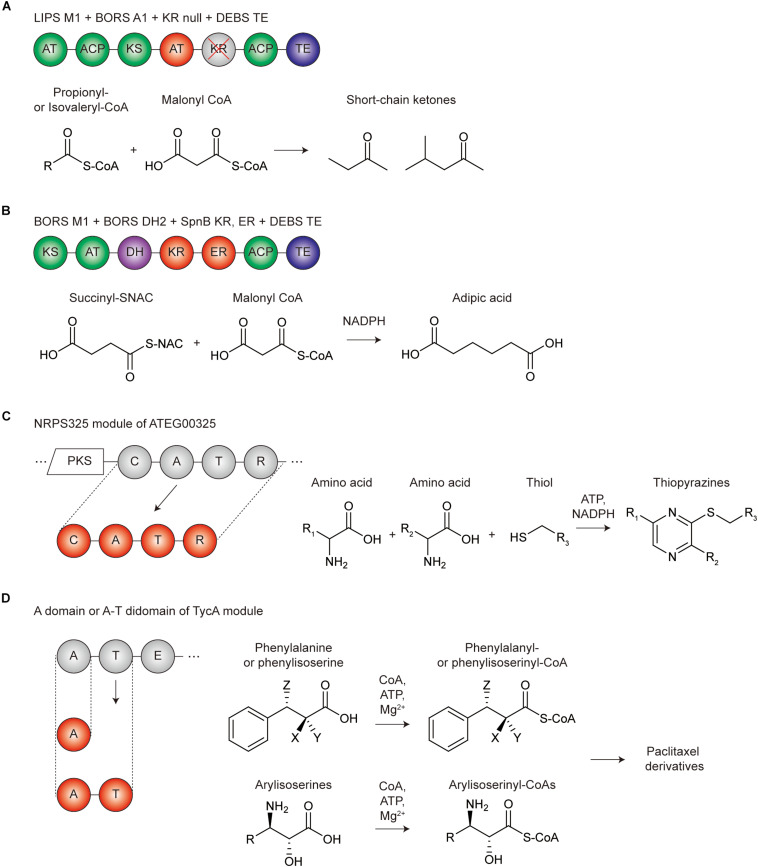
Representative repurposing examples of modular PKS and NRPS for *de novo* biosynthetic pathways. **(A)** Repurposing the PKS domains and modules for the production of short-chain ketones. Green circles are the domains in module 1 of β-lipomycin PKS (LIPS M1), red circles are the AT domains in module 1 of borrelidin PKS (BORS A1), gray circles with the red crossed line are the inactivated KR domain (KR null), and the blue circles are the TE domain of DEBS PKS. **(B)** Repurposing the PKS domains and modules for the production of adipic acid. Green circles are the domains in module 1 of borrelidin PKS (BORS M1), red circles are the KR and ER domain in SpnB module of spinosyn PKS (SpnB KR, ER), and the blue circles are the TE domain of DEBS PKS. **(C)** Repurposing the NRPS module for the production of thiopyrazines. NRPS325 module of ATEG00325 PKS-NRPS hybrid megasynthetase was isolated (red circles) to promote the reaction for the thiopyrazine production itself. **(D)** Repurposing the NRPS domain for the production of paclitaxel derivatives. The A or A-T didomain in TycA module of tyrocidine A PKS was isolated (red circles) to be repurposed for the production of phenylalanyl-, phenylisoserinyl-, arylisoserinyl-CoAs, which are the precursors of the paclitaxel derivatives; X, NH_2_ or H; Y, H or OH; Z, NH_2_ or H.

As discussed above, numerous studies have repurposed modular PKSs and NRPSs via modifications and swapping of domains and modules in the original enzyme assembly lines, mainly for the production of novel derivatives. Otherwise, domains and modules were isolated and fused to elucidate the protein–protein interactions and substrate specificities of domains with the truncated product as a proof. Although there were several combinatorial examples of domain or module fusion to form the *de novo* enzyme assembly lines, their products were generally not the purpose of the study. Recently, technical advances of the separation and fusion of domains and modules from original enzyme assembly lines, owing to numerous engineering studies, opened the way for retro-biosynthesis. Retro-biosynthesis is a *de novo* pathway design that assembles reactions in a stepwise fashion in the reverse direction of synthesis, for the desired product (Birmingham et al., 2014) (Figure 4). In this approach, the final product formation is the sole selection criterion for each reaction step that reduced the screening effort for the high-throughput combinatorial experiments. By leveraging the potential diversity of the reactions from modular PKSs and NRPSs, the potential range of chemicals produced would be countless. Furthermore, this new pathway may have advantages in terms of reaction thermodynamics and economics. Despite these potentials, recently there have only been a few examples of repurposing PKSs and NRPSs due to experimental difficulties. In this section, we highlight recent efforts of repurposing PKS and NRPS domains and modules for the production of non-natural commodity chemicals or specialty chemicals by *de novo* pathway construction.

### Redesigning Modular PKSs for Retro-Biosynthesis

Type I modular PKSs have favorable properties that functions and order of their modular catalytic domains determine the final product in a predictable manner. Most recently, an *in silico* toolkit called ClusterCAD, a computational platform for designing novel multi-modular type I PKS, has been developed and applied to several PKS repurposing studies (Eng et al., 2018). However, previous studies indicated that constructing multi-modular PKSs to produce novel chemical is still challenging (Weissman, 2016; Pang et al., 2019). The relationship between PKS module structure and acyl chain passage from one module to the next is not well understood thus, when testing the multi-modular assembly line, the acyl chain extension was frequently stalled at the middle of the synthesis process. Hence, presently, only recombination of one or two PKS modules to produce simple structured chemicals has been successfully conducted. To date, representative target molecules produced by using PKSs are divided into two categories: (i) fuels and (ii) industrial chemicals (Table 6).

**TABLE 6:** Selected examples of modular PKS and NRPS repurposing for *de novo* biosynthetic pathways.

**Category**	**Product**	**Host**	**Assembly line**	**References**
Bio-based fuel	3-hydroxy acids	*In vitro*	LipPks1 from lipomycin PKS + TE from erythromycin PKS	Yuzawa et al., 2017b
Bio-based fuel	3-hydroxy acids	*Streptomyces venezuelae*	LipPks1 from lipomycin PKS + TE from erythromycin PKS	Yuzawa et al., 2017a
Bio-based fuel	Short chain ketones	*In vitro, Escherichia coli*	AT swapped LipPks1 from lipomycin PKS + TE from erythromycin PKS + KR null	Yuzawa et al., 2017b
Bio-based fuel	Short chain ketones	*Streptomyces albus*	AT swapped LipPks1 from lipomycin PKS + TE from erythromycin PKS + KR null + truncated N-terminal linker of KAL	Yuzawa et al., 2018
Industrial chemicals	Adipic acids	*In vitro*	Reductive loop swapped BolLM-BorMod1 from borrelidin PKS + TE from erythromycin PKS	Hagen et al., 2016
Industrial chemicals	Triketide lactones	*Escherichia coli*	154 designed bimodular PKSs based on 14 modules form eight PKSs	Menzella et al., 2005
Industrial chemicals	Triketide lactones	*Escherichia coli*	54 designed trimodular PKSs	Menzella et al., 2007
Industrial chemicals	Triketide lactones	*Streptomyces coelicolor*	DEBS1 + TE from erythromycin PKS	Regentin et al., 2004
Industrial chemicals	Triketide lactones	*Streptomyces venezuelae*	DEBS1 + TE from erythromycin PKS	Yan et al., 2012
Industrial chemicals	Triketide lactones	*Saccharomyces cerevisiae*	Mod2 of DEBS1 + TE from erythromycin PKS	Mutka et al., 2006
Potentially useful chemical	Thiopyrazine	*In vitro*	Isolated NRPS module (NRPS325) of isoflavipucine PKS-NRPS megasynthetase (ATEG00325)	Qiao et al., 2011
Anticancer	Paclitaxel derivatives	*In vitro*	Isolated NRPS domain (A or A-T domain) of tyrocidine synthetase TycA module + semisynthesis	Muchiri and Walker, 2012, 2017

Petroleum-based fuels are mixtures of highly reduced carbons with varying chain length as in the case of gasoline, which is mixture of C5 to C8 hydrocarbons. To date, bio-based hydrocarbon production has majorly relied on utilizing enzymes involved in isoprenoid and fatty acid biosynthesis (Beller et al., 2015). However, the alkane and alkene produced from these systems were generated as a mixture (Beller et al., 2010; Sukovich et al., 2010; Howard et al., 2013). As a way to overcome this limitation, theoretically, PKS modules can be engineered to produce hydrocarbons with specific carbon length. Recently, several cases have been reported that resulted in the over-production of pentadecaheptaene (PDH) by expressing iterative PKS of enediyne biosynthesis SgcE and its cognate TE SgcE10 in *Escherichia coli* or *Streptomyces* (Van Lanen et al., 2008; Zhang et al., 2008). Furthermore, through optimizing the enzyme ratio of SgcE and SgcE10, PDH production was enhanced, followed by the additional chemical reduction to overproduce the pentadecane (PD) (Liu et al., 2015). Presently, there is no example of producing hydrocarbons using only refactored modular PKS, but chemical reduction steps such as hydrogenation are required after the precursor production. Conversely, short-chain ketones have been successfully produced by using only repurposed PKS. Due to the high octane number, short-chain ketones have the potential to be utilized as gasoline replacements or gasoline oxygenates (Yuzawa et al., 2018). In addition, short-chain ketones are widely used as flavors and fragrances. PKS for short-chain ketone production was designed by repurposing the first PKS module from the lipomycin PKS and linking TE from erythromycin PKS (LipPks1 + TE). Originally, LipPks1 + TE was constructed for production of various 3-hydroxy acids depending on the starter acyl-CoAs (Yuzawa et al., 2017a,b). Engineering the same PKS by changing active site serine residue of KR domain to alanine deactivated the KR function and resulted in the production of various short-chain ketones (Yuzawa et al., 2017b).

A representative industrial chemical produced by re-designed PKS is adipic acid, a monomer component used to prepare the polymer Nylon 6,6. Conventionally, bio-based production of adipic acid has been conducted by producing *cis,cis*-muconic acid, followed by chemical hydrogenation (Niu et al., 2002), or constructing β-oxidation reversal and ω-oxidation pathways, which synthesized the dicarboxylic acid from glucose or glycerol (Clomburg et al., 2015). For PKS based C6 adipic acid synthesis, a novel condensation strategy was proposed, which condenses C4 succinyl-CoA as the starter unit and C2 malonyl-CoA as the extender unit. Loading AT domain of the borrelidin PKS (BolLM) originally incorporates a *trans*-1,2-cyclo-pentanedicarboxylic acid CoA (CPDA-CoA) as the starter unit, but it has promiscuity to successfully recognize succinyl-CoA (Hagen et al., 2014, 2016). Furthermore, the first extension module of the borrelidin PKS (BorMod1) naturally incorporates malonyl-CoA. Thus, BolLM-BorMod1 linked with the TE domain from erythromycin produced 3-hydroxyadipic acid (Hagen et al., 2014). To convert 3-hydroxyadipic acid to adipic acid, further β-carbonyl processing governed by the DH and ER domains’ is required, however, BorMod1 only contains a KR domain and lacks the DH and ER domains. Therefore, the reductive loop of BorMod1 was replaced with reductive loop from other PKSs containing full tridomains, resulted in successful synthesis of adipic acid (Hagen et al., 2016). Nevertheless, further studies are still needed for *in vivo* adipic acid production using PKS, as the starter unit, succinyl-CoA, is essential for the growth of the producing host and the promiscuity of the PKS modules will decrease the titer.

As fossil resources are depleted, the bio-based eco-friendly production of transport fuels and commodity chemicals, previously been produced from these fossil resources, is becoming more common. We believe that repurposing PKS will play a large role in this field. Additionally, further mechanistic understanding of PKS domains is expected to enable the design and implementation of PKS capable of producing even chemicals that do not occur naturally.

### Redesigning Modular NRPSs for Retro-Biosynthesis

Non-ribosomal peptides are the most widely spread and structurally diverse secondary metabolites. NRPS modules are much more versatile than PKS modules in terms of the number of available substrate chemicals, including non-proteinogenic amino acids, thus the potential of producing novel chemicals by repurposing the module seems to be much higher. Nonetheless, current examples of NRPS repurposing are limited, compared to PKS, this may be due to the structural and mechanistic complexity of NRPS. In the case of PKS repurposing examples, the *de novo* pathway constructions for the useful chemical production were done by the combination of only unrelated PKS modules themselves. On the other hand, NRPS repurposing examples used a single NRPS domain or module reaction alone or combined with other non-NRPS enzymes. Thus, selected examples were discussed that showed the potential of further NRPS module repurposing.

The first example is a case that confirmed unexpected chemical production when a single NRPS module was isolated and expressed from the PKS-NRPS hybrid assembly line. Previously, novel PKS-NRPS assembly line was designed to produce tryptophan-containing preaspyridone analog by replacing the NRPS domain of PKS-NRPS megasynthetase (ApdA) of *Aspergillus nidulans*, which originally synthesizes tetramic acid preaspyridone, with the NRPS domain of cyclopiazonic acid synthetase (CpaS) of *Aspergillus flavus* (Liu and Walsh, 2009). Based on these results, the same group dissected and tested the function of a single NRPS module (NRPS325) of PKS-NRPS megasynthetase (ATEG00325) from *Aspergillus terreus*, which produces isoflavipucine. Unexpectedly, the NRPS module can synthesize thiopyrazine *in vitro*, which is largely different from the original role of the module in the parent enzyme (Qiao et al., 2011). This showed that in a multi-modular assembly line, a single NRPS module is capable of producing potentially useful chemicals and can be utilized as a part to construct *de novo* biosynthetic pathways for non-natural compound production.

Another example was the repurposing of a tyrocidine NRPS domain (A domain or A-T didomain) to the paclitaxel biosynthesis pathway to produce various paclitaxel derivatives (Muchiri and Walker, 2012, 2017). Antimitotic, anticancer paclitaxel was originally produced from *Taxus brevifolia* or the semisynthetic method, which suffered from low yields and environmentally harmful reactions, respectively. Moreover, the synthesis of the most essential precursor, amino phenylpropanoyl CoA substrates, was a major limitation as, it required protection at their amino groups before synthetic thioesterification. The authors hypothesized that A domain in tyrocidine NRPS module TycA could be used as the potential chemoselective carboxylate CoA ligase that originally had phenylalanine specificity but showed potential promiscuity. As a result, α-, β-phenylalanyl, and (2*R*,3*S*)-phenylisoserinyl CoA were successfully generated by the A domain (Muchiri and Walker, 2012). By employing 16 substituted phenylisoserines, the A-T didomain of TycA converted them to their corresponding isoserinyl CoAs, resulting in the production of docetaxel, milataxel, and various other analogs (Muchiri and Walker, 2017). These examples showed that a single NRPS domain could be integrated to other biosynthetic or semisynthetic pathways and utilized as a part for the construction of *de novo* pathways for non-natural compound production.

Owing to the natural diversity of the amino acid substrates other than acyl CoA substrates, the substrate promiscuity of the NRPS modules seems to be broader than PKS modules. Although these aspects of NRPS hindered the understanding of their reactions, further advances would increase the potential of NRPS to be repurposed for the construction of *de novo* biosynthesis pathways of useful chemicals, by fusing the NRPS modules.

## The Roadmap for Repurposing Modular PKS and NRPS

As shown above, modular PKS and NRPS have been studied with the aim of (i) engineering for the production of novel chemicals, and (ii) repurposing the domain and module reactions toward *de novo* biosynthetic pathways for the production of useful chemicals. To overcome previous limitations of these approaches for the expansion of the diversity of their products and optimization of their productivity, the systematic strategy such as the design-build-test-learn cycle should be applied. The brief explanations, applications, and perspectives of the tools for each step are discussed below.

### Design Tools

Design tools include structural, kinetic, mechanistic, and sequence-based techniques to obtain fundamental information for the experimental design of the repurposing. Specifically, the structural and kinetic knowledge is collected and sorted to the database, and bioinformatics tools are used for domain/module selection and boundary identification based on the database. First, structural biology tools were used for the modular PKS and NRPS, which are macromolecules with numerous protein–protein interactions and dynamic conformational change during biosynthesis. Thus, the structures and interactions of the domains and one or two modules have been reported for high-resolution and dynamic scale. X-ray crystallography was the most commonly used technique that provided high-resolution images at the angstrom level (Conti et al., 1997; Keatinge-Clay and Stroud, 2006; Tang et al., 2006; Buchholz et al., 2009). However, it would require the protein to crystallize which can be difficult for large structures. Additionally, the dynamic conformations and protein–protein interactions could not be inferred. To complement these limitations, conventional electron microscopy was utilized but it required additional staining, and suffered from relatively low resolution due to the deformation of flexible particles (Hoppert et al., 2001). Also, NMR spectroscopy was predominantly practiced in the structural determination of ACP and docking domains, and their conformation dynamics (Broadhurst et al., 2003; Richter et al., 2008; Lim et al., 2012). However, the size limitation of this technique hindered to be utilized for the whole module structure. Other alternative techniques also reported were the integrated utilization of X-ray crystallography and electron microscopy (Tarry et al., 2017), and Small-angle X-ray scattering (SAXS) or spectroscopic methods for the study of time-resolved conformations (Edwards et al., 2014; Alfermann et al., 2017). Recently, cryo-EM technique was applied to obtain the high-resolution and dynamic structural information of module 5 of pikromycin PKS and pentaketide-bound form of the module, but the homogeneous sample should be prepared carefully (Dutta et al., 2014; Whicher et al., 2014). By harnessing the rapid technical developments, the dynamic structures of the larger enzyme assemblies including multi-module level could be further elucidated.

In addition to the structural efforts, kinetic profiling of the modular PKS and NRPS has been vigorously utilized to measure the substrate specificity of AT and A domain, and to understand the protein–protein interactions between various domains or modules. This information is essential for the determination of the target and method for engineering. In case of the substrate specificity and affinity assays, integrated UV assay with NADPH consumption for acyltransferase activity in PKS (Lowry et al., 2013), and pyrophosphate exchange assay, pyrophosphate release assay, and hydroxylamine quenching assay for adenylation activity in NRPS have been performed (Villiers and Hollfelder, 2009; Wilson and Aldrich, 2010; Katano et al., 2013; Stanisic and Kries, 2019). The substrate specificity of AT, A, KS, and C domains in the elongation module toward growing polyketide chains or polypeptide tethered to phosphopantetheine (Ppant) arm of ACP and T domain was studied by using the mimicking molecule such as acyl- or aminoacyl-*N*-acetylcysteamine thioesters (acyl- or aminoacyl-SNACs) (Ehmann et al., 2000; Jensen et al., 2012). The studies of the protein–protein interactions commonly exploited the mechanism-based crosslinkers, inhibitors, and probes. Most of them covalently attached to the Ppant arm or other active sites of ACP or T domain. There were many examples of PKSs including phosphopantetheine analogs, photo-crosslinking benzophenone, and azide-alkyne click chemistry linkers for the understanding of KS-AT, AT-ACP, and ACP-KS interactions (Worthington et al., 2006; Ye and Williams, 2014; Ye et al., 2014; Ladner and Williams, 2016). The interactions were quantified by means of radioisotopic transfer assay, thermodynamics heat using calorimetry, and fluorescence, or MS-based techniques. For NRPS, mechanism-based inhibitors such as 5′-*O*-sulfamoyladenosine (AMS) and adenosine vinylsulfonamide (AVS), azide-alkyne click chemistry linkers, and biotin probes were mainly used for the studies of NRPS A-T interactions (Finking et al., 2003; Sundlov et al., 2012; Ishikawa and Kakeya, 2014; Stanisic and Kries, 2019). These crosslinkers were versatile tools for the pretreatment of structural crystallization (Mitchell et al., 2012). As various tools have been used for the kinetic profiling, it might be better to compare between them for the reproducibility, or to establish the universal standard or tool.

Based on the information obtained from the structural and kinetic studies, computational approaches and bioinformatics would be required to design the detailed processes of the engineering of PKS and NRPS enzymes, or pathways (Medema and Fischbach, 2015; Alanjary et al., 2019). For example, the protein or DNA sequence boundaries of the domain, module, and linker for the swapping could be determined from the database of genome, protein, and smBGC (Marchler-Bauer et al., 2015; Blin et al., 2019; Sayers et al., 2020). In the case of the absence of the structural and kinetic profiling data, comparative analysis method was mainly used including sequence alignment tools, database search tools, and evolutionary analysis tools (Navarro-Munoz et al., 2020). Homology modeling tools such as I-TASSER, MODELLER, and SWISS-MODEL were frequently used to predict the structure of PKS or NRPS by comparative analysis with the reported similar structures for the engineering design (Roy et al., 2010; Waterhouse et al., 2018; Bitencourt-Ferreira and de Azevedo, 2019). Lastly, the integration of the enzyme reactions with their substrates, products, protein sequence, and gene sequence was applied to the automated pathway design pipeline tool of the combinations of reactions for the final products (Carbonell et al., 2014; Eng et al., 2018). Along with the structural and kinetic profiling tools, bioinformatic tools should be integrated and continuously updated to provide the universal, reproducible, and versatile strategy to involve the full pipeline of database search, comparative analysis, homology modeling, and experimental design.

### Build Tools

Build tools are technical tools for preparing DNA parts, proteins, and hosts to test the experimental design from above. In recent decades synthetic biology tools have been rapidly developed, enabling large-scale, combinatorial, and high-throughput engineering (Winn et al., 2016; Lee et al., 2019). Despite these advanced tools, they are not frequently used for PKS and NRPS engineering as yet. In case of the genetic manipulation tools, the DNA sequence fragments of the domain and module for the engineering were obtained from the native source, mostly by traditional PCR amplification and restriction enzyme digestion. The rapid and inexpensive cost of DNA synthesis and the highly efficient, accurate DNA digestion tools such as CRISPR-Cas9 would replace the conventional restriction enzyme-based cloning method (Lee et al., 2015; Hughes and Ellington, 2017). Moreover, homologous recombination based DNA assembly tools such as Gibson assembly, linear-linear homologous recombination (LLHR), and yeast TAR cloning would be more efficient for the large size and numbers of DNA fragments than traditional ligation methods (Fu et al., 2012; Jiang et al., 2015; Lee et al., 2015). Site-directed mutagenesis for the PKS and NRPS enzymes would be also easier by exploiting CRISPR-Cas9 system and the base-editors, even for *in vivo* genome editing (Komor et al., 2016). Directed evolution of synthetic libraries for the modular PKS and NRPS are feasible with these genetic manipulation tools. Furthermore, *in vitro* expression techniques such as protein purification tools and precursor synthesis tools as well as *in vivo* expression techniques in the heterologous host, such as promoter refactoring and metabolic pathway engineering, would simultaneously be developed with genetic manipulation tools (Chen et al., 2007; Weber et al., 2015; Winn et al., 2016).

### Test Tools

Test tools were used to quantitatively measure the novel derivatives from engineering, or the products from novel pathways, to analyze the chemical structure of the products and to screen the desired clone from the libraries. The most widely applied tools for the quantitative measurement of the products were high-throughput mass spectrometry techniques that were also used for the screening and chemical analysis (Krug and Muller, 2014; Boiteau et al., 2018). By integration with other techniques such as liquid chromatography, the separation and evaluation of exact molecular weight, amount, and chemical moieties for the product could be interrogated. In many cases, the screening step is the bottleneck, which requires rapid and accurate selection of the desired clone among the extensive libraries. Leveraging the biological activity of the product for screening was the traditional screening assay logic that involves an inhibition zone assay for antibacterial activity, colorimetric assay for pigment product, and growth assay for the auxotrophic strain of the product such as siderophore (Balouiri et al., 2016; Cleto and Lu, 2017; Wehrs et al., 2019). As these methods were limited to specific products and the small size of the libraries, the universal and high-throughput screening strategies such as yeast surface display combined with FACS (Zhang et al., 2013), Ppant ejection strategy detecting the intermediate of the rate-limiting step by protease (Meluzzi et al., 2008), and biosensor development for the PKS and NRPS product such as the macrolide biosensor MphR (Kasey et al., 2018), along with mass spectrometry, would be a more favorable method.

### Design-Build-Test-Learn (DBTL) Cycle for Modular PKS and NRPS Repurposing

**FIGURE 5:**
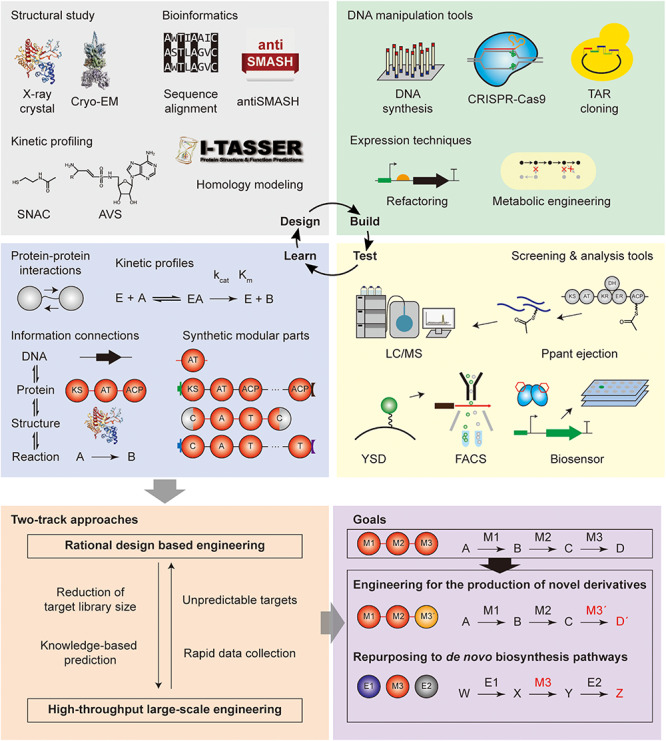
Roadmap for repurposing modular PKS and NRPS. Design-build-test-learn cycle with the tools for each step was illustrated.

Learning from the design-build-test steps of modular PKS and NRPS repurposing would be; (i) the updated information of the protein–protein interactions and the kinetic profiles to the substrate of the enzymes, (ii) the appropriate splicing or engineering sites for the construction of synthetic parts, (iii) the rationale for the selection of compatible sets, (iv) the integration of the information of cognate reactions, sequences, substrate, and products, and (v) the reversible connections between the combination of reactions and the final product for retro-biosynthesis (Bayly and Yadav, 2017). Based on the current understanding, iterative DBTL cycles would ultimately achieve the goal of novel chemical productions as well as novel pathway generation (Figure 5).

## Conclusion

We reviewed selected examples of modular PKS and NRPS repurposing for the generations of novel chemicals and pathways, followed by the roadmap in view of the synthetic biological DBTL cycle. The most important lesson from the repurposing examples was the requirement of careful considerations for dissecting the complex protein–protein interactions, despite their functionality in modular fashion. Therefore, the rational engineering design should continuously be improved by the characterization of the modular PKS and NRPS from the large amount of structural, kinetic, and genetic studies, to predict and understand the results. In parallel, the massive and rapid approaches, such as directed evolution and combinatorial strategy along with high-throughput screening, should be widely adopted to find the unpredictable factors from rational engineering. Technical advances for build tools could overcome the previous limitations for the identification of the exchangeable unit and the case-by-case optimization. Ultimately, the two approaches would complement each other by the reducing library size by rational design and the reflection of the updated information learned from the non-rational large-scale strategy. An automated pipeline of the modular PKS and NRPS repurposing for retro-biosynthesis is expected to greatly expand the reservoir of the bio-active compounds.

## Author Contributions

B-KC and SH conceived the study. SH, NL, and B-KC drafted the manuscript. SH, NL, SC, BP, and B-KC contributed to the final version of the manuscript.

## Conflict of Interest

The authors declare that the research was conducted in the absence of any commercial or financial relationships that could be construed as a potential conflict of interest.
